# A Randomized Controlled Pilot Trial of a Behavioral Physical Activity Intervention for Pregnancy Hyperglycemia

**DOI:** 10.1155/jp/7485092

**Published:** 2025-11-20

**Authors:** Samantha F. Ehrlich, Bethany R. Hallenbeck, Nikki B. Zite, Kimberly B. Fortner, Alissa Paudel, Hollie A. Raynor, Scott E. Crouter, Jill M. Maples

**Affiliations:** ^1^Department of Kinesiology, Recreation, and Sports Studies, University of Tennessee, Knoxville, Tennessee, USA; ^2^Kaiser Permanente Northern California Division of Research, Pleasanton, California, USA; ^3^Department of Obstetrics & Gynecology, College of Medicine, University of Tennessee Health Science Center, Knoxville, Tennessee, USA; ^4^Center for Women and Infants, University of Tennessee Medical Center, Knoxville, Tennessee, USA; ^5^Department of Nutrition, University of Tennessee, Knoxville, Tennessee, USA

**Keywords:** behavioral intervention, gestational diabetes, hyperglycemia, pregnancy, walking

## Abstract

**Introduction:**

This randomized controlled pilot trial evaluated a behavioral physical activity (PA) intervention for individuals with pregnancy hyperglycemia and explored the feasibility of a fully powered efficacy trial.

**Materials and Methods:**

The pilot trial sought to enroll and randomize participants to a 5-week-long behavioral PA intervention that promoted walking or stepping (i.e., in place or around a small area) versus a general wellness intervention (that provided no information on PA, diet, or metabolism), both delivered remotely via weekly, 10–20-min-long counseling sessions with a lifestyle coach. Participants (*N* = 20) completed surveys, including the Pregnancy Physical Activity Questionnaire, and wore ActiGraph CentrePoint watches for 7 days at baseline and at follow-up. Nineteen participants (95%) completed follow-up study visits. A subset (85%) had neonatal anthropometric measurements due to pandemic-related restrictions.

**Results:**

One hundred and twenty individuals were screened, with 54% (*n* = 65) meeting eligibility criteria and receiving physician approval to contact; 26% of the eligible enrolled, were randomized, and completed a baseline visit. Ninety percent of those randomized to the PA intervention (*n* = 9) completed it, rating the PA intervention as excellent (56%) or very good (44%). The PA intervention mitigated late pregnancy declines in self-reported walking and running activity (follow-up minus baseline: 0.22 MET h/week [95% CI −0.41, 0.84] in the PA intervention vs. −0.70 [−1.31, −0.10] in controls), and there was the suggestion of improvements in neonatal birthweight for gestational age *Z*-score and subscapular skinfold.

**Conclusion:**

Findings suggest that the behavioral PA intervention promoting unsupervised, moderate-intensity walking or stepping, which could easily be delivered in conjunction with clinical medical nutrition therapy, was acceptable. The intervention may mitigate late pregnancy declines in moderate-intensity PA and remains to be investigated in a full-scale randomized controlled efficacy trial.

**Trial Registration:**

ClinicalTrials.gov identifier: NCT06125704

## 1. Introduction

Pregnancies complicated by gestational diabetes mellitus (GDM) are at increased risk for adverse birth outcomes, including a large-for-gestational-age neonate and shoulder dystocia. These risks increase with increasing maternal glucose below the levels used to diagnose GDM [1] and have been observed in individuals with gestational glucose intolerance (GGI) [2, 3]. Treatment for GDM begins with medical nutrition therapy, weight management, and physical activity (PA) [4]. Levels of PA are typically suboptimal among reproductive-aged and pregnant individuals and decline over the course of pregnancy [5–7]. Although specific numeric targets and recommendations are available to support medical nutrition therapy (i.e., a personalized meal plan) and gestational weight gain (i.e., weekly weight gain targets, per the 2009 Institute of Medicine Guidelines), guidance on PA counseling is currently lacking [8].

This randomized controlled pilot trial sought to evaluate the acceptability of a behavioral PA intervention [9, 10] promoting moderate-intensity walking or stepping (i.e., walking in place or around a small area) in individuals with GGI or GDM. The intervention included planning and goal setting for unsupervised PA, in anticipation of future translation to the clinical setting and delivery in conjunction with medical nutrition therapy. This pilot trial also sought to demonstrate the feasibility of a future, fully powered randomized controlled efficacy trial to evaluate the behavioral PA intervention in our setting.

## 2. Materials and Methods

This parallel, one-to-one randomized controlled pilot trial took place at the University of Tennessee Medical Center (UTMC) in Knoxville and the University of Tennessee, Knoxville. Eligibility criteria included the following: 18–40 years of age, singleton viable pregnancy with low suspicion for congenital abnormality or aneuploidy, English speaking, planning to remain in the area, and a 1-h plasma glucose greater than or equal to 130 mg/dL on a 50-g glucose screening test after 24 weeks of gestation. Exclusion criteria included the following: pregestational diabetes, contraindications to exercise, current smoker, illicit drug use, and current use of medication known to alter metabolism or treat polycystic ovarian syndrome.

Potentially eligible patients were referred to the study by clinic staff, who evaluated inclusion and exclusion criteria through electronic medical records. Final physician approval for recruitment was then obtained (NBZ and KBF). Eligible patients were contacted via text message and phone. Potential participants attended a meeting, during which study staff explained the protocol in detail and answered questions. Informed consent was then obtained from all subjects.

The UTMC IRB approved the trial (#4547), which was registered at clinicaltrials.gov (registration first released on 24/12/2019). The CONSORT statement extension to randomized pilot and feasibility trials [11] guided reporting. The trial initially launched in December 2019 but closed in March 2020 due to the COVID-19 pandemic. It relaunched in August 2020, after being redesigned to accommodate remote trial participation and intervention delivery. Assessment of maternal glucose via masked continuous glucose monitoring devices, originally a primary outcome, became optional [12], and the target sample size was reduced to 20. In-person contact resumed when it was deemed appropriate by the UTMC IRB and the trial's safety officer. A flow chart with eligibility and recruitment metrics is therefore presented for December 2020 through September 2021, the period unaffected by research restrictions.

At the height of the pandemic, materials were left on participants' doorsteps prior to study visits conducted remotely; otherwise, staff met participants at a location of their choosing. At baseline and follow-up, participants completed paper surveys and had their weight measured. They wore a CentrePoint Insight Watch, a research-grade activity monitoring device by ActiGraph, continuously for 7 days on the dominant wrist.

Randomization occurred upon completion of the baseline study visit and was stratified by GDM versus GGI. As recommended by the American Diabetes Association [13], GDM could be diagnosed by either the one-step strategy (a 75-g oral glucose tolerance test [OGTT] with plasma glucose meeting or exceeding: 92 mg/dL for fasting, 180 mg/dL for 1 h, or 153 mg/dL for 2 h) or the two-step strategy (a [nonfasting] 50-g, 1-h glucose screening test meeting or exceeding 130 mg/dL, followed by a 100-g, 3-h OGTT with two or more plasma glucose measurements meeting or exceeding 95 mg/dL for fasting, 180 mg/dL for 1 h, 155 mg/dL for 2 h, and 140 mg/dL for 3 h). Those with GGI only had a 1-h plasma glucose ≥ 130 mg/dL on the 50-g screening test, measured at 24+ weeks' gestation (i.e., followed by an OGTT that did not meet the GDM criteria above).

The randomization sequence was determined in SAS (SFE) and blocked (size 4) to ensure equal sample sizes between groups. Allocation was concealed by an outside investigator and revealed to intervention staff upon completion of the baseline study visit. Intervention staff informed the participants of their group assignment. Data assessors (BR and JMM) were blinded to group status.

### 2.1. Interventions

The behavioral PA counseling intervention was based upon previously successful lifestyle interventions for pregnancy and postpartum weight management, developed for the clinical setting [14, 15], and centered on Bandura's social cognitive theory [16] and the transtheoretical model [17]. Behavior change techniques included (but were not limited to) the following: self-monitoring of and feedback on behavior, goal setting, review of behavior goals, problem-solving, and action planning [9].

The *program goal* was to achieve at least 30 min of walking or stepping (i.e., walking in place or around a small area) at moderate intensity (i.e., ~100 steps/min) every day. An overview of the program and safety considerations was reviewed at the first session. Remaining core sessions covered the following: keeping walking or stepping interesting and challenging yourself (i.e., by increasing the frequency, intensity, and/or time spent walking or stepping), handling challenges, celebrating success, and staying motivated. Motivational interviewing techniques [18, 19] helped participants set reasonable, specific, and achievable *individual goals* at each session: a duration (i.e., minutes) and frequency (i.e., days of the week) of walking or stepping and, potentially, a step count (i.e., a proxy for intensity). Individual goals often differed from the program goal in an effort to meet the participants “where they are.” The interventionist met with participants weekly by Zoom, for 15–20-min intervention sessions, which included five core sessions and the option to continue for up to three additional sessions, as desired. Progress toward individual goals was reviewed at each session, feedback was provided, and new/repeat individual goals were set.

The control group received a general wellness intervention (i.e., five weekly, 15–20-min-long Zoom sessions). The following topics were addressed: postpartum contraception, maternal immunizations, infant immunizations, car seat safety and planning for your hospital stay, and safe sleep and skin to skin. No information on PA was provided to the control group (i.e., neither encouragement for PA nor instructions to limit PA).

### 2.2. Outcomes

#### 2.2.1. PA

The CentrePoint Insight Watch data included 7-day observation periods at baseline and follow-up and were processed in RStudio 2022.02.3+492. Periods in which the watches were not worn, as identified by the algorithm of Choi et al., were removed [20–22], and observation days with at least 600 min of device wear were retained for analyses. The TwoRegressions [23] R package provided minute-by-minute estimates of the metabolic equivalent of task (MET) and coefficients of variation (CVs) for each timestamped datapoint. Datapoints with MET ≥ 3.0 and CV ≤ 21.2% for devices worn on the right wrist or ≤ 19.4% for the left wrist were identified as continuous walk/run activity of moderate to vigorous intensity [23]. Minutes of moderate-to-vigorous-intensity continuous walk/run activity (“walk/run activity” hereafter) and minutes of all moderate-to-vigorous-intensity activities were then summed per day.

A modified version of the Pregnancy Physical Activity Questionnaire (PPAQ) [24, 25] provided self-reported estimates of PA during the past month, both at baseline and follow-up. The present study focused on the PPAQ questions within the sports and exercise domain: PA of moderate to vigorous intensity that is intentional for health, wellness, or to increase fitness and results in energy expenditure beyond the demands of everyday living. It included 10 PPAQ activities of moderate intensity (range: 3.2–6 METs for walking, swimming, etc.) plus two PPAQ activities of vigorous intensity (6.5 and 7 METs for walking quickly up hills and jogging, respectively), performed for fun or exercise. Response options include ranges for the amount of time spent in the activity (e.g., none, < 1/2 h/day, 1/2 to almost 1 h/day, and 1 to almost 2 h/day). The minimum value of the selected range (i.e., duration and frequency of that activity) was multiplied by the activity's intensity, expressed in MET, to derive estimates of the volume of each activity [25]. Summing these provided an overall estimate of the volume of sports and exercise activity in MET hours per week. Analyses also examined PPAQ questions within the sports and exercise domain specific to walking (three questions) or jogging (one question), as these activities were the focus of the intervention.

#### 2.2.2. Neonatal Anthropometrics

Neonatal weight was measured with a Seca 354 pediatric scale, length with a Seca 210 infant measuring mat, and head, abdominal, and arm circumferences with a Seca 201 measuring tape. Birthweight for gestational age was based on the sex- and gestational age–specific birthweight distributions of the 2017 US natality files [26]. Skinfolds at the flank, thigh, tricep, bicep, and subscapular were measured with Harpenden calipers on the right side of the body. Neonatal fat mass was estimated with the equation of Catalano et al. [27]. All neonatal measurements were taken by a single data assessor (JMM) within 72 h of delivery, with the exception of the first neonate to be measured after pandemic-related in-person research closures, who was measured 5 days after birth. Neonatal measurements were taken in duplicate, when possible, and then averaged.

### 2.3. Statistical Analyses

Analyses were intention-to-treat. Mean daily minutes spent in walk/run activity and MVPA (i.e., device-based measures), as well as mean MET hours per week in the sports and exercise domain overall and just walking and jogging (i.e., questionnaire-based measures), were compared between groups with Proc Mixed in SAS 9.4. The DDFM = KR option was used to calculate Kenward–Roger degrees of freedom (due to the small sample size) and compound symmetry or the variance component structure for the covariance structure. All models were adjusted for GGI versus GDM status. Models of the device-based measures included repeated statements for observation day number by study visit. Minutes per day of walk/run activity were log-transformed so the residuals would approximate a normal distribution. The model for total MVPA included additional adjustment for device wear-time (i.e., by day and study visit). Models for the questionnaire-based measures included repeated statements for the study visit. For the neonatal outcomes, PA intervention versus control group differences were examined in Proc Mixed, adjusting for GGI versus GDM status. The approximate sample size needed to detect significant between-group differences was computed using PASS 2023, Version 23.0.1.

## 3. Results

**Figure 1:**
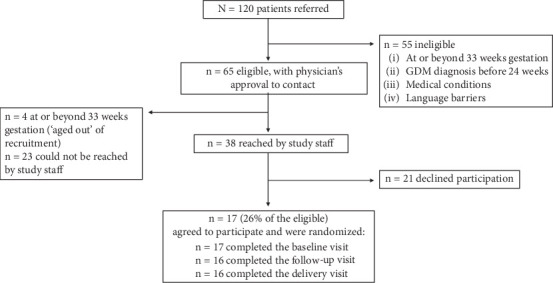
Pregnant patients with hyperglycemia referred for potential participation in a randomized controlled pilot trial of a behavioral physical activity intervention.

Referral, eligibility, and recruitment data for December 2020 through September 2021 are presented in Figure 1. During this period, a total of 120 individuals were referred to study staff, with 55 (46%) found to be ineligible, primarily because they were either at or beyond 33 weeks of gestation upon referral (*n* = 23) or they were diagnosed with GDM before 24 weeks (*n* = 17). Of the 65 eligible individuals, 38 (58%) were reached by study staff and invited to participate, 21 declined participation, and 17 (26% of the eligible) agreed to participate, completed a baseline visit, and were randomized to an intervention. Of these 17 participants, 16 had neonatal anthropometric measurements taken by study staff. No serious adverse events were reported during the trial.

Across the entire recruitment period (December 2019 to August 2021), 20 individuals agreed to participate, completed a baseline visit, and were randomized. Of these, 95% (*n* = 19) provided follow-up data approximately 6 weeks later, including one participant who delivered early and thus did not have PAM data at follow-up and another participant with damaged PAM data at follow-up (though both had self-reported PA data at follow-up). Baseline characteristics are displayed in Table 1; groups were approximately balanced, except for education and employment status.

Ninety percent (*n* = 9) of participants randomized to the PA intervention completed all five core intervention sessions; 78% (*n* = 7) opted to continue meeting with the lifestyle coach for at least one additional session (range: one to three additional sessions). Participants (*n* = 9) rated the PA intervention as excellent (56%) or very good (44%) and rated their lifestyle coach as excellent (89%) or very good (11%). They reported that talking with the lifestyle coach was either very (44%) or moderately (56%) helpful and that the length of the intervention sessions and number of sessions offered were appropriate.

The PA data are presented in Table 2. Participants randomized to the control group were more active at baseline than those assigned to the PA intervention. The PA intervention group added 0.90 min/day of device-assessed walk/run activity (95% CI 0.53, 1.54), while the control group reduced their walk/run activity by 1.58 min/day (95% CI 0.90, 2.80) between baseline and follow-up (*p* = 0.14). The between-group difference for change in device-assessed daily minutes of MVPA also did not attain statistical significance (*p* = 0.58). Based on the daily minutes of device-assessed total MVPA, 44 participants (22 per group) would be needed to detect a statistically significant interaction between study visit and group (with an effect size for the interaction of 0.44, a power of 0.80, and an alpha of 0.05).

In the PPAQ data, the between-group difference for change in walking and jogging activity attained statistical significance (*p* = 0.04): Those in the PA intervention reported no change in moderate-to-vigorous-intensity walking and jogging activity over time (i.e., activity volume at baseline minus at follow-up of 0.22 MET h/week [95% CI −0.41, 0.84]), while those in the wellness intervention reported a decrease of 0.70 MET h/week (95% CI −1.31, −0.10) (Table 2). No between-group difference was detected for change in overall, self-reported moderate-to-vigorous-intensity sports and exercise activity (*p* = 0.24).

The neonatal data are presented in Table 3. Due to pandemic-related restrictions, 17 neonates had weight, length, circumference, and skinfolds measured by study staff; all neonates had data on infant birthweight and sex in the electronic medical record. Between-group differences in the neonatal outcomes were all in the hypothesized direction (i.e., the PA intervention group's infants were smaller as compared to the control group's infants) but did not attain statistical significance. There was a suggestion of between-group differences for weight-for-gestational-age *Z*-score and subscapular skinfolds (both *p* = 0.07). Based on the subscapular skinfolds' measurements presented in Table 3, 32 participants (16 per group) would be needed to detect a statistically significant (with a power of 0.80 and an alpha of 0.05) between-group difference in subscapular skinfolds.

## 4. Discussion

This randomized controlled pilot trial demonstrated that the behavioral PA intervention was well received and that a fully powered randomized controlled efficacy trial is feasible in our setting. The present pilot trial relied on passive recruitment strategies (i.e., medical center clinic staff referred potentially eligible individuals to the study staff) and still achieved 26% participation among eligible individuals, which is comparable to other lifestyle intervention trials in pregnant people [15]. The behavioral PA intervention also may have mitigated late pregnancy declines in self-reported moderate-to-vigorous-intensity walking and jogging activity in individuals with GGI or GDM [28–32]. In order to more efficiently recruit for a future full-scale randomized controlled trial in this setting, we have since successfully added in-person recruitment strategies (e.g., staff physically present in the lab for recruitment during patients' 100-g, 3-h OGTTs) to allow for a larger pool of potentially eligible participants. Accommodations made for remote participation and intervention delivery in the present pilot trial should be reexamined; the added flexibility eased participant burden and, if the PA intervention is found to be efficacious, could simplify clinical implementation in the future. However, a recent systematic review and meta-analysis of lifestyle interventions for gestational weight gain management in women with overweight or obesity reported that in-person interventions may be more efficacious than remote (i.e., eHealth or mHealth) interventions, noting that the data were currently limited and insufficient for the PA components in particular [33]. Social disadvantage increases the risks of adverse pregnancy outcomes [34]; thus, it is important that the intervention modality is widely accessible and does not have the unintended consequence of furthering inequalities in health access.

Those randomized to the behavioral PA intervention did not decrease self-reported walking and running over time, while those randomized to the control group did. Self-reported walking and running decreased by 0.70 MET h/week in the control group, but this small decrease is unlikely to have a significant clinical impact. For example, for a moderate-intensity activity like brisk walking at 3.0 mi/h (i.e., a 3.5 MET activity), 0.70 MET h represents only approximately a 12-min/week decrease in self-reported walking and running (i.e., 0.70 MET h/3.5 METs = 0.2 h × 60 min). Further, those randomized to the behavioral PA intervention were less active at baseline, more highly educated, and more often employed part-time or not looking for work as compared to controls. These sociodemographic characteristics tend to be associated with higher participation in PA during pregnancy and better overall health, but with a lower activity level at baseline, those in the intervention did not have the opportunity to decrease their activity level over time to the same degree as those in the control group. Increased knowledge and social desirability bias among those randomized to the behavioral PA intervention may have also contributed, at least in part, to this finding.

Small-sized trials are more likely to suffer from between-group differences in participant characteristics; indeed, the present pilot trial's major limitation is its small sample size of 20 individuals. Pilot trials are not typically powered to detect significant differences between groups. Flat rules of thumb typically recommend 60–100 subjects per group for binary outcomes and 12–35 per group for continuous outcomes [35–39]. Our sample size was constrained by the logistical challenges of the COVID-19 pandemic, and thus, we were left with a less precise effect estimate for planning the main trial. However, obtaining these data, despite the COVID-19 pandemic, is a strength. The present pilot trial included both self-reported and device-based measures of moderate-to-vigorous-intensity *walking*, the specific mode of activity promoted by the behavioral PA intervention, which is a strength. It also enabled us to assess key processes needed for the future success of the main trial (e.g., prevalence of patients meeting eligibility criteria, recruitment rate, and protocol compliance rates), as well as resources (e.g., the amount of time required to complete the study visits and intervention sessions and staff time to perform all required tasks) and data management (e.g., matching data from different sources) [40].

There was the suggestion that the PA intervention led to decreases in neonatal weight-for-gestational-age *Z*-score and subscapular skinfolds at birth, consistent with the literature suggesting that infant body composition could be influenced by higher volumes of PA performed in mid to late pregnancy [28]. Neonates with higher subscapular skinfolds are more common in GDM [30] and likely contribute to the high prevalence of shoulder dystocia in this population. A follow-up analysis of 698 participants from the UK Pregnancies Better Eating and Activity Trial (UPBEAT), conducted among pregnant individuals with obesity, found that the pregnancy lifestyle intervention, which targeted maternal diet and PA, reduced subscapular skinfold thickness at 6 months of age by 5% compared with infants in the standard care arm [32]. Causal mediation analyses revealed that the lifestyle intervention's effects on the infants' subscapular skinfold thickness were partially mediated by changes in dietary fat and energy intake at 28 weeks of gestation and gestational weight gain; mediation by PA during pregnancy was not investigated [32].

### 4.1. Practical Applications

Treatment for pregnancy hyperglycemia typically begins with clinical counseling for lifestyle modification [4]. Both medical nutrition therapy and weight management counseling efforts are supported by numeric targets (i.e., ranges for grams of carbohydrate per meal or snack and a prepregnancy BMI–specific weekly rate of gestational weight gain, respectively). The present behavioral PA intervention similarly relied on numeric targets and goal setting for unsupervised, moderate-intensity walking or stepping, thereby leveraging behavioral counseling strategies already in use with this population in the clinical setting and promoting a nearly universally available mode of activity. Should the behavioral PA intervention be found efficacious in a full-scale randomized controlled trial, it could easily be delivered in conjunction with medical nutrition therapy.

Personalized meal plans developed in medical nutrition therapy incorporate personal and cultural food preferences, as well as logistical considerations (e.g., when the patient will be at work versus at home that day), to maximize patient adherence. Finding time was the most often cited barrier to PA in the present pilot trial (and has previously been reported [41]); thus, goal setting for walking or stepping on specific days, at specific times, similar to personalizing meals to an individual's upcoming schedule, emerged as a key strategy to helping participants meet their PA goals.

## 5. Conclusions

The present randomized controlled pilot trial demonstrated that the behavioral PA intervention is acceptable and that a fully powered randomized controlled efficacy trial is feasible in this setting. Future investigation of the behavioral PA intervention's efficacy in maintaining or increasing PA levels across late pregnancy and improving maternal and neonatal outcomes, as well as the behavioral PA intervention's cost-effectiveness and potential for implementation in conjunction with medical nutritional therapy, represents exciting opportunities for clinical translation.

## Figures and Tables

**Table 1:** Pilot trial participant characteristics (*N* = 20), Department of Obstetrics and Gynecology, Graduate School of Medicine, University of Tennessee Medical Center, Knoxville, Tennessee, 2020–2021.

	**Physical activity intervention (** **n** = 10**)**	**General wellness intervention (** **n** = 10**)**
	**Mean (SD)**	**Mean (SD)**
Age (years)	32.5 (4.6)	32.5 (3.7)
Gestational age at baseline (weeks)	31.2 (1.4)	29.9 (1.9)
Gestational age at follow-up (weeks)	36.4 (0.6)	35.9 (0.9)
Prepregnancy BMI (kg/m^2^)	25.0 (6.7)	25.1 (3.2)
	*n* (%)	*n* (%)
GDM	4 (40)	4 (40)
White	8 (80)	8 (80)
Primipara	6 (60)	5 (50)
Married	10 (100)	10 (100)
Education		
Technical/trade school, some college, or 2-year college degree	2 (20)	2 (20)
4-year college graduate	0 (0)	6 (60)
Postgraduate degree	8 (80)	2 (20)
Employment		
Full-time (≥ 35 h/week)	5 (50)	7 (70)
Part-time (< 35 h/week)	4 (40)	0 (0)
Not employed outside the home, not looking for work	1 (10)	3 (30)

**Table 2:** Group mean (95% confidence interval) device- and questionnaire-based physical activity metrics by timepoint and change in the physical activity metrics (*N* = 20), Department of Obstetrics & Gynecology, Graduate School of Medicine, University of Tennessee Medical Center, Knoxville, Tennessee, 2020–2021.

	**Physical activity intervention**		**General wellness intervention**		**p** ** value**
	***n***	**Mean (95% CI)**	***n***	**Mean (95% CI)**	
MVPA by device (minutes per day)					
Walk/run activity only^ab^					
26–33 weeks' gestation	10	9.21 (5.58, 15.03)	10	13.20 (7.54, 22.87)	
34–37 weeks' gestation	9	10.18 (6.23, 16.78)	8	8.33 (4.76, 14.44)	
Change^c^		0.90 (0.53, 1.54)		−1.58 (−2.80, −0.90)	0.14
All MVPA^d^					
26–33 weeks' gestation	10	75.3 (49.5, 101.2)	10	77.2 (51.4, 103.0)	
34–37 weeks' gestation	9	69.7 (43.2, 96.2)	8	62.6 (34.8, 90.4)	
Change^c^		−5.6 (−29.0, 17.8)		−14.6 (−39.4, 10.2)	0.58
Moderate-to-vigorous-intensity sports & exercise by questionnaire (MET hours per week)					
Walking and running only^b^					
26–33 weeks' gestation	10	0.55 (0.00, 1.09)	10	1.53 (0.99, 2.07)	
34–37 weeks' gestation	9	0.76 (0.20, 1.33)	9	0.82 (0.28, 1.37)	
Change^c^		0.22 (−0.41, 0.84)		−0.70 (−1.31, −0.10)	0.04
All moderate-to-vigorous-intensity sports & exercise^b^					
26–33 weeks' gestation	10	7.73 (2.73, 12.74)	10	15.53 (10.53, 20.54)	
34–37 weeks' gestation	10	8.80 (3.53, 14.08)	10	11.13 (6.13, 16.14)	
Change^c^		1.07 (−5.69, 7.82)		−4.40 (−10.93, 2.12)	0.23

Abbreviations: MET, metabolic equivalent of task; MVPA, moderate-to-vigorous-intensity physical activity.

^a^Outcome was log-transformed for modeling; the back-transformed results are presented here.

^b^Model adjusted for gestational glucose intolerance versus gestational diabetes.

^c^Change = follow‐up − baseline.

^d^Model adjusted for gestational glucose intolerance versus gestational diabetes and device wear-time.

**Table 3:** Neonatal measurements (*N* = 20), Department of Obstetrics & Gynecology, Graduate School of Medicine, University of Tennessee Medical Center, Knoxville, Tennessee, 2020–2021.

	**Physical activity intervention**	**General wellness intervention**	**Adjusted** ^**a**^ ** group mean difference (SE)**	**p** ^**a**^
	**Mean (95% CI)**	**Mean (95% CI)**		
	**(** **n** = 10**)**	**(** **n** = 10**)**		
Weight (kg)	3.07 (2.71, 3.44)	3.32 (3.01, 3.62)	−0.24 (0.22)	0.30
Weight-for-gestational-age *Z*-score^b^	−0.87 (−1.51, −0.24)	−0.064 (−0.70, 0.57)	−0.81 (0.43)	0.07
	(*n* = 7)	(*n* = 10)		
Length (cm)	49.55 (48.02, 51.08)	49.83 (48.55, 51.11)	−0.28 (0.93)	0.77
Circumference (cm)				
Head	33.99 (32.82, 35.16)	35.21 (34.23, 36.19)	−1.22 (0.71)	0.11
Waist	32.51 (30.59, 34.43)	34.24 (32.64, 35.85)	−1.74 (1.17)	0.16
Arm^c^	10.12 (9.41, 10.84)	10.23 (9.63, 10.82)	−0.10 (0.44)	0.82
Skinfolds^c^ (mm)				
Flank	3.52 (3.02, 4.02)	3.74 (3.32, 4.16)	−0.22 (0.30)	0.48
Thigh	6.21 (5.16, 7.25)	6.94 (6.07, 7.81)	−0.73 (0.64)	0.27
Tricep	4.72 (4.07, 5.37)	5.14 (4.60, 5.68)	−0.42 (0.40)	0.31
Subscapular	4.37 (3.65, 5.08)	5.24 (4.64, 5.84)	−0.87 (0.44)	0.07
Bicep	3.77 (3.35, 4.20)	4.05 (3.69, 4.40)	−0.27 (0.26)	0.31
Neonatal fat mass^d^ (g)	1198.81 (1055.83, 1341.79)	1293.45 (1173.97, 1412.92)	−94.64 (87.17)	0.30

^a^Adjusted for gestational glucose intolerance versus gestational diabetes.

^b^Based on the sex- and gestational age–specific birthweight distributions of the 2017 US natality files, which provide cut points for births occurring at 22–42 weeks' gestation.

^c^Measured on the right side of the body.

^d^Estimated by the equation of Catalano et al. Anthropometric estimation of neonatal body composition, *Am J Obstet Gynecol*. 1995 Oct;173[4]:1176-81.

## Data Availability

The data analyzed are not publicly available due to the small size of the sample and concerns that individual privacy could be compromised. Data (stripped of all personal identifiers) will be available from the corresponding author upon reasonable request, with permission of the University of Tennessee Medical Center IRB and following entrance into a Data Use Agreement (DUA).

## Nomenclature

GDM: gestational diabetes mellitus
GGI: gestational glucose intolerance
MET: metabolic equivalent of task
PPAQ: Pregnancy Physical Activity Questionnaire
MVPA: moderate-to-vigorous-intensity physical activity
OGTT: oral glucose tolerance test
PA: physical activity
RCT: randomized controlled trial
UTMC: University of Tennessee Medical Center
